# Removal of As(V) from aqueous solution using modified Fe_3_O_4_ nanoparticles

**DOI:** 10.1098/rsos.220988

**Published:** 2023-01-25

**Authors:** Yuling Zhao, Hao Shi, Ze Du, Jinlong Zhou, Fangyuan Yang

**Affiliations:** ^1^ College of Resources and Environment, Xinjiang Agricultural University, Urumqi, Xinjiang 830052, People's Republic of China; ^2^ College of Hydraulic and Civil Engineering, Xinjiang Agricultural University, Urumqi, Xinjiang 830052, People's Republic of China; ^3^ College of Mathematics and Physics, Xinjiang Agricultural University, Urumqi, Xinjiang 830052, People's Republic of China

**Keywords:** nanomaterials, Fe_3_O_4_, arsenic, adsorption

## Abstract

The removal of arsenic contamination from the aqueous environment is of great importance in the conservation of the Earth's water resources, and surfactants are a promising material used to modify magnetic nanoparticles to improve adsorption properties. Therefore, it is important to develop efficient and selective adsorbents for arsenic. Surface modification of Fe_3_O_4_ was carried out using anionic, cationic and zwitterionic surfactants to obtain composite Fe_3_O_4_@SDS, Fe_3_O_4_@CTAB, Fe_3_O_4_@SNC 16 and Fe_3_O_4_@NPC 16 (collectively referred to as Fe_3_O_4_@surfactants). The synthesized composite Fe_3_O_4_@surfactants magnetic nanoparticles were characterized by XRD, TEM and FTIR. The As(V) removal characteristics of the composite magnetic nanoparticles from the aqueous solution were evaluated by adsorption batch experiments which indicated the possibility of effective application of the surfactant-modified Fe_3_O_4_ magnetic nanoparticles for the removal of As(V) from aqueous solution. The adsorption equilibrium of the composites was reached in 30 min and the kinetic data followed the pseudo-second-order model. Langmuir equation could represent the adsorption isotherm data very well. Moreover, under the identical conditions, Fe_3_O_4_@CTAB showed maximum capacity of adsorption for As(V) (55.671 mg g^−1^), with its removal efficiency being much higher than that of the other composites. In addition, the Fe_3_O_4_@surfactants composite magnetic nanoparticles retained 93.5% of its initial arsenic removal efficiency even after re-using it five times. The mechanism of arsenic adsorption by Fe_3_O_4_@surfactants composite magnetic nanoparticles was proved to be complexation via electrostatic attraction, which was mainly innersphere in nature.

## 1. Introduction

Polluted water having toxic heavy metals is a global problem [1]. Among various heavy metals, arsenic contamination has been a global menace. Exposure towards polluted water containing arsenic over a period of time would result in cancer of various organs including lung, skin, blader, liver and renal. Co-precipitation, filtration, adsorption, ion exchange, membrane dialysis and biomass remediation [2–4] are the presently followed techniques applied for decontamination of drinking water with respect to arsenic.

Adsorption-based technologies are the most promising amidst various treatment methods mentioned above as a result of their efficiency, simple operation and cost-effective nature [3–5]. Various adsorbents like zeolites [6,7], clays [8–12], activated carbon [13], biomaterials [14], metal oxides [15], zero-valent iron [16] and neutralized red mud [17] have found applications for arsenic removal from polluted water. However, low adsorption capacity and difficulty in separation and regeneration of these adsorbents have restricted their practical application [18]. Magnetic nano-materials are exceptional and environmentally friendly due to their directional motion in a magnetic field, which allows them to be separated from solution by simple magnets after performing adsorption [19]. Applicability of magnetic nano- and micro-particles for the removal of metals from wastewater has been reviewed by Ngomsik *et al*. [20]. Among various magnetic nanoparticles, magnetite [12] and maghemite [13] nanoparticles were studied for the removal of Cr(V), while Fe_3_O_4_ magnetic nanoparticles bound with chitosan found application in Cu(II) removal [14].

When in contact with oxygen for a long period of time, Fe_3_O_4_ magnetic nanoparticles tend to lose their magnetism, and, from a colloidal point of view, the nanoscale particles form a less stable system that determines their agglomeration [21]. Various methods and chemicals have recently been studied for the modification of magnetic iron oxide nanoparticles in order to prevent the oxidation of magnetic nanoparticles [22–27]. Agglomerated particles have less specific surface area and interfacial free energy resulting in decreased activity and loss of unique property of the nanoparticles [28,29]. To prevent the agglomeration phenomenon of nanoparticles, the immobilization of iron trioxide in three-dimensional porous carbon nanofibres (3DPCNF) networks to form a host–guest structure is an effective method to prevent agglomeration, and iron-manganese-layered double hydroxide (MnFe-LDH) metal oxides were prepared on the surface of MnFe_2_O_3_-immobilized 3DPCNF (MnFe-LDH/MnFe_2_O_3_@3DNF) by Poudel *et al*. [30,31] and showed good adsorption performance for As(III) with a maximum adsorption capacity of 504.54 mg g^−1^.

Various stabilizers like carboxylic acids [32], surfactants [33] and polymers [34] have proven to be effective in preventing agglomeration of nanoparticles. Beyond a certain concentration, surfactant forms micelles in solution. Thus, surface modification of iron nanoparticles using micelles can enhance their dispersion into groundwater or soils by preventing their aggregation [35,36]. Previous studies have found that the aggregation of nanoparticles is inhibited to great extent via modification with cetyltrimethylammonium bromide (CTAB) [37–39]. For example, Maleki *et al*. [40] proved sodium dodecyl sulfate (SDS) micelle-coated Fe_3_O_4_/SiO_2_ nanoadsorbent to be an effective solid phase adsorbent for crystal violet from aqueous solution. Abdel Ghafar *et al*. [41] reported an effective treatment method of polluted water containing toxic metals or azo dyes via zwitterionic surfactant CAPB-modified natural clay. The removal efficiency of magnetic nanoparticles for the removal of pollutants has been found to be enhanced via different structural and surface modifications.

This manuscript reports the synthetic procedure of Fe_3_O_4_ magnetic nanoparticles via co-precipitation route along with surfactant-based surface modification of the synthesized magnetic nanoparticles, to probe the arsenic removal properties of nano-materials modified with different electrical surfactants. Different types of surfactants like anionic (SDS), cationic (CTAB) and zwitterionic (palmityl sulfobetaine, SNC 16 and Miltefosine, NPC 16) were employed to modify magnetite (Fe_3_O_4_) nanoparticles. FTIR, XRD and TEM techniques were employed to investigate the shape and structure of the synthesized nanoparticles. The adsorption efficiency of Fe_3_O_4_ magnetic nanoparticles and Fe_3_O_4_@surfactants magnetic nanoparticles (for different surfactants) was compared for the removal of As(V). Various parameters were optimized to obtain the best adsorption condition, while applying various kinetic and equilibrium adsorption models. Finally, mechanistic insight towards the promotional effect of surfactant-coated Fe_3_O_4_ for the adsorption of As(V) was also looked into.

## 2. Materials and methods

### 2.1. Materials

**Figure 1.**
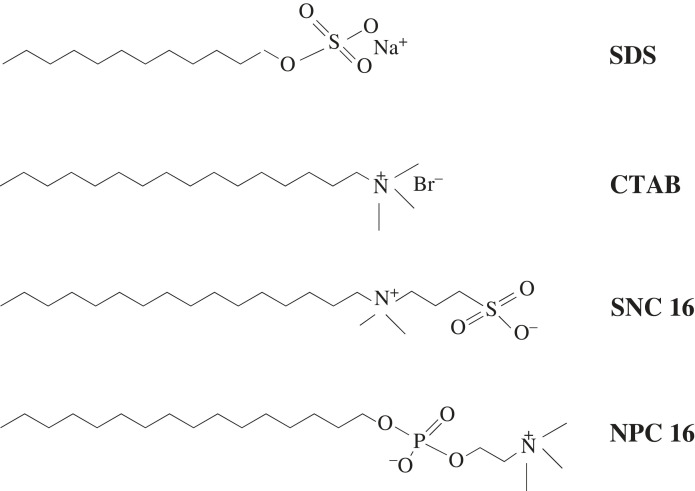
Molecular formulae of SDS, CTAB, SNC 16 and NPC 16.

The surfactants used in this study included SDS, CTAB, SNC 16 and NPC 16. Their corresponding molecular formulae are shown in figure 1. CTAB was purchased from Sigma-Aldrich Company. SDS, SNC 16 and NPC 16 were used from TCI Company. Various iron salts (FeCl_3_ · 6H_2_O and FeSO_4_ · 7H_2_O) and ammonia water (NH_3_·H_2_O) were obtained from Tianjin Zhiyuan Chemical Reagent Co., Ltd. All the solutions were prepared from deionized water. All the above reagents were of analytical purity and did not require further purification.

### 2.2. Synthesis of Fe_3_O_4_@surfactants magnetic nanoparticles

Four different surfactant-coated Fe_3_O_4_ nanosorbent materials were prepared by co-precipitation method. First, 2.78 g FeSO_4_ · 7H_2_O and 5.4 g FeCl_3_ · 6H_2_O [*n*(Fe^3+^) ∶ *n*(Fe^2+^) = 2 : 1] were weighed in four portions in four double-necked flasks with 100 ml of deionized water and dissolved by ultrasonication, followed by oxygen removal employing a vacuum pump. Then the system was heated to 90°C in a water bath. Next, 0.1 mol l^−1^ of SDS, CTAB, SNC 16 and NPC 16 with 10 ml volume of each were separately added quickly to each reaction system after injecting 10 ml of NH_3_·H_2_O.

The solution was heated under stirring condition for 30 min and then was allowed to settle, which was followed by cooling to allow the formation of a precipitate. The precipitate was centrifuged at 9000 r.p.m. for 5 min, collected and washed twice using the alternate steps of deionized water-centrifugation and anhydrous ethanol-centrifugation, which was followed by vacuum drying at 60°C for 12 h. Then, the final black solid powder products of Fe_3_O_4_@SDS, Fe_3_O_4_@CTAB, Fe_3_O_4_@SNC 16 and Fe_3_O_4_@NPC 16 were obtained.

### 2.3. Characterization

The crystalline phase of Fe_3_O_4_@SDS, Fe_3_O_4_@CTAB, Fe_3_O_4_@SNC 16 and Fe_3_O_4_@NPC 16 was characterized using XRD (Bruker D2, Germany) with Cu K*α* radiation and a step size of 0.02°. Morphology of the nanoparticles was characterized using transmission electron microscopy (JEM 2100F, Japan). The FTIR spectra of the nanoparticles were measured in the range of 400–4000 cm^−1^ using an infrared spectrometer (Scientific Nicolet iS5, America) employing KBr pellet. Magnetic behaviour of the particles was studied using a vibrating sample magnetometer (MPMS-XL-7, USA) in the varying magnetic field strengths of ±3000 magnetic moments. An X-ray electron spectrometer (ESCALAB 250XI, America) was used to determine the element type, valence and phase ratio of the sample.

### 2.4. Adsorption test

The adsorption kinetics of As(V) were measured from a solution with pH adjusted to 6 employing HCl or NaOH. The feed solution contained 5 mg l^−1^ As(V) into which Fe_3_O_4_@surfactants particles with a concentration of 1 g l^−1^ were added. The time of equilibration was varied from 2, 5, 10, 20, 40, 60, 90, 120 to 150 min employing a magnetic stirrer. After sampling, the concentration of As(V) in the samples was measured using an atomic fluorescence spectrophotometer (PF6-3, Beijing Purkinje General Instrument Co., Ltd) after passing the samples through 0.45 µm membrane filter.

The adsorption isotherm experiments were carried out by varying the initial concentration of As(V) from 0.1, 0.5, 1, 3, 5, 7 to 10 mg l^−1^. The other experimental conditions were as discussed above.

### 2.5. Desorption and regeneration studies

To explore the possibility of multiple use of the adsorbents, adsorption–desorption cycle for each adsorbent was studied for five cycles. In each cycle, 5 mg l^−1^ of As(V) solution was contacted with 1 g l^−1^ of different adsorbents for 1 h on a magnetic stirrer, and the desorption of As(V) was carried out by contacting with 200 ml sodium hydroxide solution having concentration 1 mol l^−1^ for 30 min. After completing the desorption process, washing of the adsorbent was carried out with deionized water containing 0.01 mol l^−1^ hydrochloric acid until the solution become neutral. Finally, the Fe_3_O_4_@SDS, Fe_3_O_4_@CTAB, Fe_3_O_4_@SNC 16 and Fe_3_O_4_@NPC 16 nanoparticles were vacuum dried in an oven at 60°C for 2 h after collecting the nanoparticles with a handheld magnet for the next cycling experiments.

## 3. Results

### 3.1. Characterization of Fe_3_O_4_@surfactants nanoparticles

**Figure 2.**
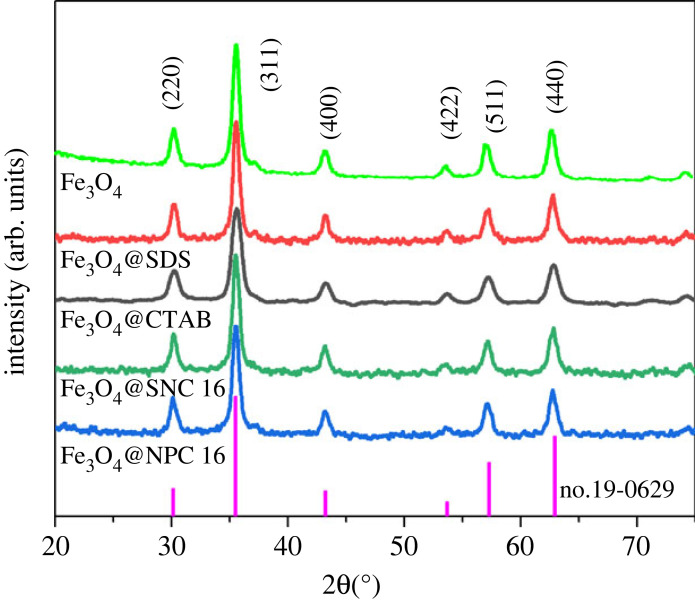
XRD spectra of Fe_3_O_4_, Fe_3_O_4_@SDS, Fe_3_O_4_@CTAB, Fe_3_O_4_@SNC 16 and Fe_3_O_4_@NPC 16.

The crystal structures of the synthesized Fe_3_O_4_@SDS, Fe_3_O_4_@CTAB, Fe_3_O_4_@SNC 16 and Fe_3_O_4_@NPC 16 nanoparticles were evaluated employing XRD measurements. As shown in figure 2, diffraction peaks were observed at 2*θ* values of 30.1°, 35.5°, 43.1°, 53.4°, 57.0° and 62.6° which could be assigned to the lattice planes of (220), (311), (400), (422), (511) and (440), respectively, of magnetite (JPCDS no. 19-0629). Overall, these results suggest the presence of cubic spinel magnetite structure for all the types of magnetic nanoparticles and also point out the prevention of oxidation of the magnetic nanoparticles due to the coatings of the surfactants. Moreover, sharp and narrow attributes of the peaks suggest highly crystalline and pure nature of the Fe_3_O_4_ nanoparticles after surfactant coating [42–44].

### 3.2. Morphological analysis

**Figure 3.**
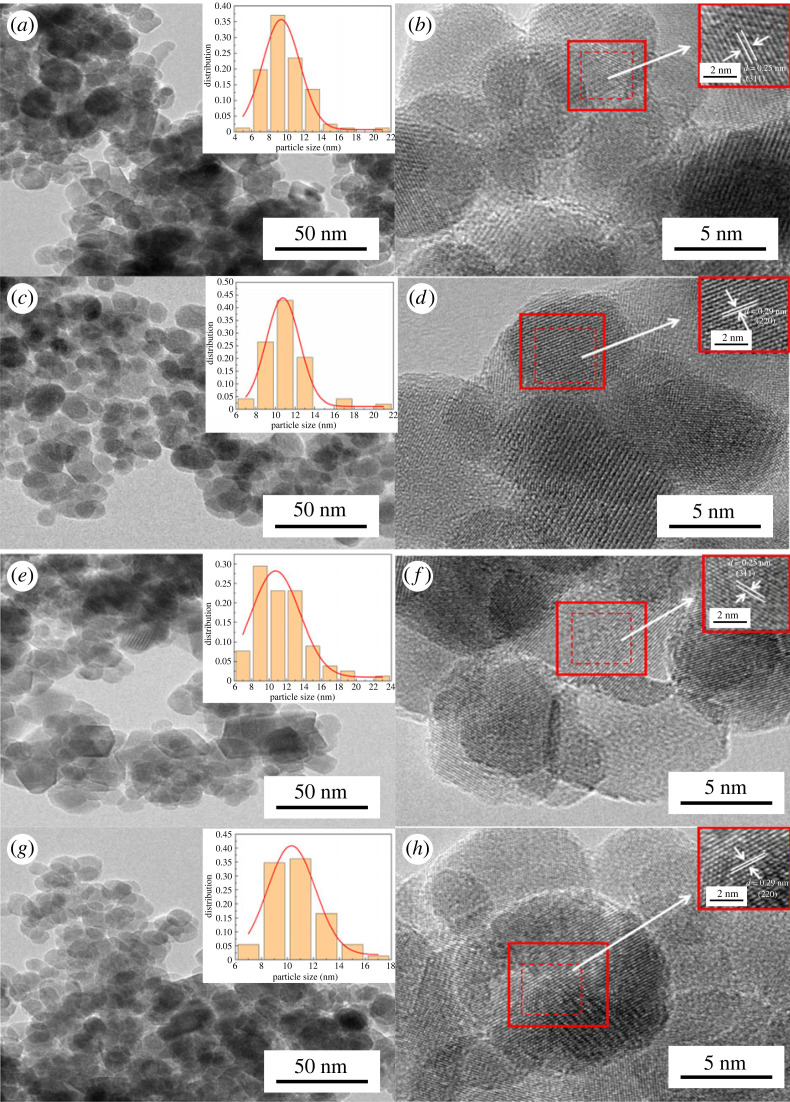
TEM particle size distributions (*a*), (*c*), (*e*), (*g*) and lattice stripes (*b*), (*d*), (*f*), (*h*) of nanoparticles Fe_3_O_4_@SDS, Fe_3_O_4_@CTAB, Fe_3_O_4_@SNC 16 and Fe_3_O_4_@NPC 16.

The TEM morphologies of Fe_3_O_4_@surfactants magnetic nanoparticles are shown in figure 3. Figure 3 shows that the nano-Fe_3_O_4_ cores indicated by dark colour are surrounded by a micellar shell of surfactants, along with the presence of slight agglomeration [45]. The TEM images of Fe_3_O_4_ modified by SDS, CTAB and zwitterionic surfactant show the presence of spherical-like shapes having an average diameter of less than 20 nm. This size range of Fe_3_O_4_ indicates superparamagnetic behaviour. This behaviour indicates that Fe_3_O_4_ magnetic nanoparticles become magnetized and show magnetic properties in the presence of an external magnetic field, and, when the magnetic field is removed, they lose their magnetic behaviour. This feature can be used for the recycling of nano-sized Fe_3_O_4_ materials. For further evaluation, the magnetic hysteresis loops of the as-synthesized Fe_3_O_4_@surfactant magnetic nanoparticles were measured for confirming their magnetic characteristics. And from (*b*), (*d*), (*f*) and (*h*) in figure 3, the lattice stripe spacing of SDS and SNC 16 were found to be 0.25 nm, corresponding to the (311) crystal plane observed from the XRD, and the lattice stripe spacing of CTAB and NPC 16 were found to be 0.29 nm, corresponding to the (220) crystal plane observed from the XRD, thus confirming the single crystal behaviour of the synthesized Fe_3_O_4_ samples.

### 3.3. FTIR spectrum

**Figure 4.**
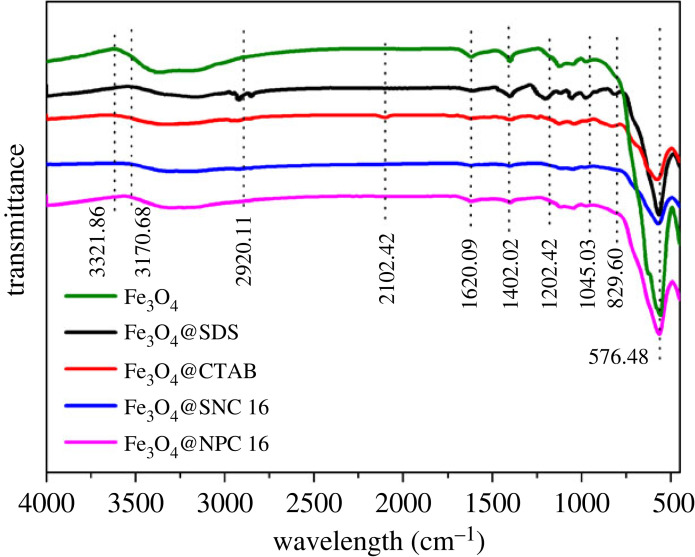
FTIR spectra of Fe_3_O_4_, Fe_3_O_4_@SDS, Fe_3_O_4_@CTAB, Fe_3_O_4_@SNC 16 and Fe_3_O_4_@NPC 16.

FTIR spectra of the as-prepared surfactant-coated Fe_3_O_4_ magnetic nanoparticles were recorded for better identification of various functional groups attached to Fe_3_O_4_. Figure 4 shows FTIR spectra of the Fe_3_O_4_@SDS, Fe_3_O_4_@CTAB, Fe_3_O_4_@SNC 16 and Fe_3_O_4_@NPC 16, which clearly show the presence of peaks in the range of 565.78 to 576.48 cm^−1^ corresponding to the strong Fe-O absorption band. The peaks at 1402.02 and 1202.42 cm^−1^ for Fe_3_O_4_@SDS are characteristic frequencies of -OSO_3_- group of the SDS, while the asymmetric stretching vibrations of -CH_2_- of SDS results in the adsorption peak at 2920.11 cm^−1^. Thus, FTIR analysis proves the presence of SDS coating in the Fe_3_O_4_@SDS nanoparticles. Moreover, the stretching vibrations of -C-CH_2_ result in the peaks at 1620.09 cm^−1^ [35], while the peak at 3321.86 cm^−1^ could be due to the amino groups [19]. This proves the successful anchoring of CTAB or zwitterionic surfactant onto the surface of Fe_3_O_4_ magnetic nanoparticles.

The above analysis clearly showed that the surfactants were successfully coated on the surface of Fe_3_O_4_ for promoting hydrate formation and the coated Fe_3_O_4_ exhibited superparamagnetism which aids in the recycling of the materials.

### 3.4. Magnetic properties of Fe_3_O_4_@surfactants nanoparticles

**Figure 5.**
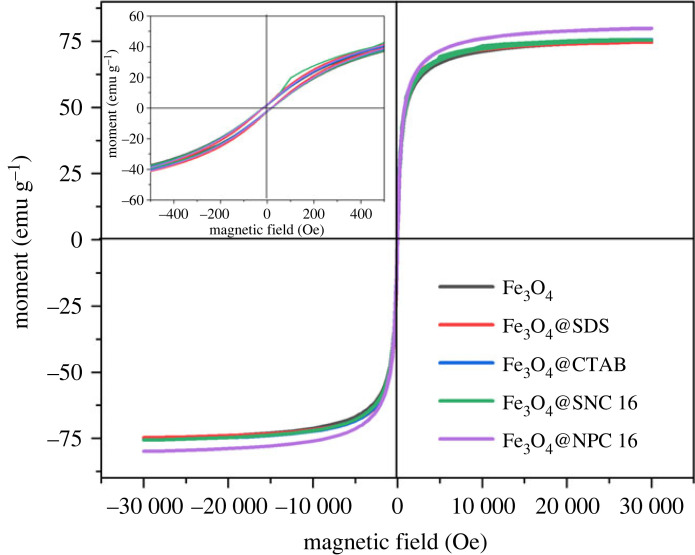
Magnetic hysteresis loop of Fe_3_O_4_, Fe_3_O_4_@SDS, Fe_3_O_4_@CTAB, Fe_3_O_4_@SNC 16 and Fe_3_O_4_@NPC 16 of nanoparticles.

Room-temperature magnetic hysteresis curves of Fe_3_O_4_, Fe_3_O_4_@SDS, Fe_3_O_4_@CTAB, Fe_3_O_4_@SNC 16 and Fe_3_O_4_@NPC 16 were measured by vibrating sample magnetometry (VSM) (figure 5). A hysteresis loop as evident from the close-up view presented in the inset of figure 5 appears for all the curves. The saturation magnetizations (*Ms*) and magnetic remanence (*Mr*) values of Fe_3_O_4_@SDS, Fe_3_O_4_@CTAB, Fe_3_O_4_@SNC 16 and Fe_3_O_4_@NPC 16 were 74.72 and 2.43, 75.73 and 2.12, 75.62 and 2.49, 79.82 and 2.28 emu g^−1^, respectively, while the respective values of coercivity (*Hc*) were 17.57, 17.61, 20.10 and 18.16 Oe. Superparamagnetic natures of all the materials were indicated from the absence of any obvious remanence or coercivity. Surface coatings by non-magnetic surfactants can be the reason behind slightly lower *Ms* values of the magnetic nanoparticles in comparison with the bare Fe_3_O_4_ (79.60 emu g^−1^) [23]. The above discussion indicated very good superparamagnetic behaviour for the synthesized surfactant-coated Fe_3_O_4_ magnetic nanoparticles, which will be beneficial in recovering the magnetic nanoparticles with the aid of an external magnetic field and then dispersing the separated Fe_3_O_4_ magnetic nanoparticles in water with the aid of ultrasound. As shown in electronic supplementary material, figure S1, all the nanoparticles have excellent dispersion behaviour and magnetic recovery in water.

### 3.5. Adsorption mechanisms

XPS measurements of the Fe_3_O_4_@surfactants magnetic nanoparticles before and after adsorption of As(V) were carried out in order to understand the adsorption mechanism of the magnetic nanoparticles adsorbent.

**Figure 6.**
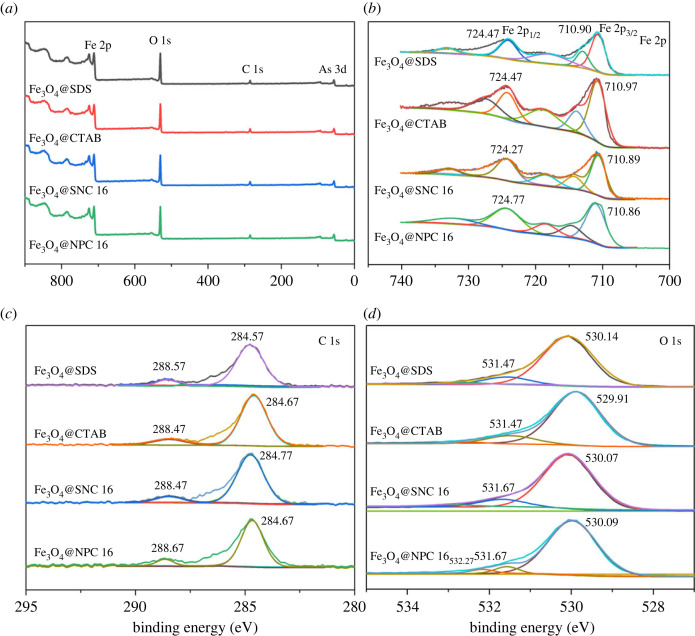
XPS spectra of synthesized surfactant-modified magnetic nanoparticles after arsenic adsorption: (*a*) XPS survey spectra; (*b*) Fe 2p; (*c*) C 1s; (*d*) O 1s.

The survey spectra of Fe_3_O_4_@surfactants after adsorption of As(V) are shown in figure 6*a*. The peaks at 45, 284, 530 and 710 eV can be attributed to As 3d, C 1s, O 1s and Fe 2p, respectively. The presence of peak corresponding to the presence of As-O bonds indicates the adsorption of As(V) on Fe_3_O_4_@surfactants magnetic nanoparticles.

The figure 6*b* shows the presence of two peaks appearing at 724 and 710 eV corresponding to Fe 2p_1/2_ and Fe 2p_3/2_, respectively. The presence of two distinct peaks for Fe 2p in the XPS spectra can be both attributed to Fe(III) and Fe(II) [46].

Figure 6*c* shows the C 1s plot after the 284.8 eV standard C correction. The C 1s XPS spectrum of Fe_3_O_4_ consisted of two components which are attributable to the appearance of C-C (284.57, 284.67, 284.77 and 284.67 eV) and C=O (288.57, 288.47, 288.67 and 286.77 eV) groups [47].

The three peaks in the O 1s spectrum appearing at binding energies of 530, 531 and 532 eV (figure 6*d*) are corresponding to that of lattice oxygen (O_2_) in the metal oxide, hydroxyl group (-OH) and adsorbed water (H_2_O), respectively [48]. As can be seen from the electronic supplementary material, slight reduction in the binding energy takes place, which could be originating from the electron donation from O 1s leading to the formation of As-O bonding and thus suggesting that surface complexation sites could be playing important roles during As(V) adsorption process. It can be inferred from the obtained results that the surface complexation between functional groups and heavy metals is complemented by the electrostatic attraction between O atoms and heavy metal ions [49].

## 4. Adsorption properties of Fe_3_O_4_@surfactants nanoparticles

### 4.1. Adsorption kinetics

Fitting of As(V) adsorption kinetic data was carried out employing both the pseudo-first-order (equation (4.1)) and pseudo-second-order (equation (4.2)) models and the obtained results are shown in table 1. Both the equations can be linearly represented as given below.

**Table 1.** Adsorption kinetics parameters for As(V) adsorption on Fe_3_O_4_@surfactants magnetic nanoparticles.

	first-order equation			second-order equation			*E_a_* (kJ mol^−1^)
	*K_1_* (min^−1^)	*q_e_* (mg g^−1^)	*R^2^*	*K_2_* (g (mg min)^−1^)	*q_e_* (mg g^−1^)	*R^2^*	
Fe_3_O_4_@SDS	0.579	4.670	0.475	0.189	4.888	0.814	75.450
Fe_3_O_4_@CTAB	1.720	4.966	0.612	2.494	4.988	0.923	107.698
Fe_3_O_4_@SNC 16	0.778	4.802	0.687	0.346	4.928	0.935	66.953
Fe_3_O_4_@NPC 16	0.793	4.843	0.778	0.363	4.962	0.979	62.277

Pseudo-first-order,

4.1 $$\ln(q_{e}-q_{t})=\ln q_{e}-k_{1}t.$$

Pseudo-second-order,

4.2 $$\frac{t}{q_{t}}=\frac{1}{k_{2}q_{e}^{2}}+\frac{t}{q_{e}},$$

where *t* is the equilibration period (min), *q_t_* and *q_e_* are the amount of As(V) adsorbed at time *t* and at equilibrium (mg g^−1^), *k*_1_ (min^−1^) is the pseudo-first-order rate constant and *k*_2_ (g (mg min)^−1^) is the pseudo-second-order rate constant. The obtained plots showed better fitting for the pseudo-second-order model as indicated by the *R*^2^ values which are closer to 1, and thus suggesting chemisorption between As(V) and the Fe_3_O_4_@surfactants magnetic nanoparticles could be the rate-limiting step [50].

Arrhenius equation (equation (4.3)) [51,52] was employed to calculate the activation energy (*E_a_*, kJ mol^−1^) of the adsorption process as given below,

4.3 $$\ln k_{2}=\ln A-\frac{E_{a}}{RT},$$

where *k*_2_ is the pseudo-second-order rate constant (g (mg min)^−1^), *A* is a constant, *R* and *T* are the universal gas constant (8.314 J (K mol)^−1^) and temperature (K), respectively.

**Figure 7.**
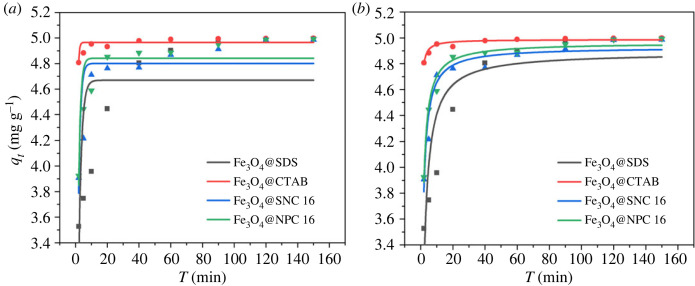
Pseudo-first-order (*a*) and pseudo-second-order (*b*) kinetics model for As(V) adsorption on Fe_3_O_4_@surfactants (pH 6, initial concentration 5 mg l^−1^, adsorbent dosage 1 mg ml^−1^, temperature 298 K, 2.5 h).

Depending on the value of *E_a_*, the adsorption process can be physical or chemical in nature. The energy of activation for physical adsorption reactions is small (ranging from 5 to 40 kJ mol^−1^). Stronger interaction forces result in higher activation energies for chemical adsorption reactions (ranging from 40 to 800 kJ mol^−1^) [53]. In the temperature range of 298 to 308 K, the *E_a_* values for all the four Fe_3_O_4_@surfactants magnetic nanoparticles were found to be greater than 40 kJ mol^−1^, thus suggesting chemisorption probably is the dominant mechanism for the adsorption of As(V) onto surfactant-modified magnetic nanoparticles figure 7.

### 4.2. Adsorption isotherm

**Figure 8.**
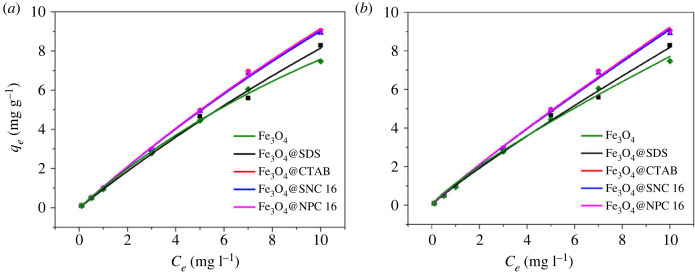
Langmuir (*a*) and Freundlich (*b*) adsorption isotherm models for As(V) adsorption on Fe_3_O_4_@surfactants (pH 6, adsorbent dosage 1 mg ml^−1^, temperature 298 K, contact time 1 h).

For devising an adsorption system with specific aim, equilibrium adsorption capacity is the most significant parameter which can be obtained from the adsorption isotherms. Two traditional isotherm models, Langmuir and Freundlich isotherms were applied to fit the experimentally obtained results for different Fe_3_O_4_@surfactants magnetic nanoparticles. Langmuir model is used to describe monolayer adsorption on surfaces with a finite number of identical locations. Freundlich isotherm model is applicable to highly inhomogeneous surfaces, and the lack of adsorption isotherms forming saturated plateaus may indicate a multi-layer adsorption mechanism [54]. Various adsorption isotherms for As(V) are presented in figure 8. The pH value of the feed during the isotherm experiment was maintained at 6 based on our earlier experiments [55]. The two isotherm models can be depicted as follows:

Langmuir model,

4.4 $$q_{e}=\frac{K_{L}q_{m}C_{e}}{1+K_{L}C_{e}},$$

Freundlich model,

4.5 $$q_{e}=K_{F}C_{e}^{\frac{1}{n}},$$

where, *q_m_* and *q_e_* are the amount of As(V) adsorbed at time *t* and at equilibrium (mg g^−1^), respectively. *K_L_* is defined as the Langmuir adsorption constant (l mg^−1^), *C_e_* is the equilibrium feed As(V) concentration (mg l^−1^), *K_F_* is the Freundlich constant related to adsorption capacity and *n* is another constant related to affinity.

Different parameters obtained from both the isotherm models are presented in table 2. As per the obtained correlation constants, the obtained experimental data can be satisfactorily explained based on both the models (*R*^2^ > 0.99). However, the Langmuir model was found to be fitting slightly better than the Freundlich model, which is in conformity with the results obtained by Su *et al*. and Pan *et al*. [48,56]. Better fitting of the Langmuir model for the four Fe_3_O_4_@surfactants magnetic nanoparticles indicates relative homogeneity of the surface with respect to the functional groups and the adsorption process being limited from the monolayer coverage. Adsorption may occur as a monolayer on uniform and equivalent adsorption sites [54]. In addition, the maximum adsorption capacities obtained from the Langmuir model were 51.039, 55.671, 52.362 and 54.589 mg g^−1^ for magnetic nanoparticles modified by SDS, CTAB, SNC 16 and NPC 16, respectively. While the maximum adsorption of As(V) by the unmodified Fe_3_O_4_ was 25.223 mg g^−1^, the surfactant modification increased the adsorption of As(V) by Fe_3_O_4_. Maximum adsorption capacity (*q_m_*) of 55.671 mg g^−1^ was obtained for the Fe_3_O_4_@CTAB magnetic nanoparticles composite. The above results indicate maximum adsorption capacity of As(V) was obtained when Fe_3_O_4_ was modified with CTAB. Strong complex formation between the positively charged CTA^+^ (cationic part of CTAB) present on the surfaces of Fe_3_O_4_ and negatively charged As(V) anions may be the reason behind this [57]. More significantly, there was a significant improvement for As(V) adsorption capacity of Fe_3_O_4_@CTAB compared with that of pure Fe_3_O_4_ [58]. The adsorption capacity of Fe_3_O_4_@CTAB for As(V) was higher than that of Jin's report [57] and can be attributed to the concentration of CTAB for modification which we choose above the average data. In addition, the removal rates of Fe_3_O_4_@SDS, Fe_3_O_4_@CTAB, Fe_3_O_4_@SNC 16 and Fe_3_O_4_@NPC 16 for As(V) were above 80% at an initial concentration of 10 mg l^−1^, 82.88%, 91.77%, 89.21% and 90.16%. Therefore, all the prepared materials could be used for the removal of arsenic from groundwater.

**Table 2.** Estimated isotherm parameters for As(V) adsorption on Fe_3_O_4_@surfactants magnetic nanoparticles.

	Langmuir			Freundlich		
	*q_m_* (mg g^−1^)	*K_L_* (l mg^−1^)	*R^2^*	1/*n*	*K_F_* (mg g^−1^) (l mg^−1^)^1/*n*^	*R^2^*
Fe_3_O_4_	25.223	0.043	0.998	0.832	1.137	0.994
Fe_3_O_4_@SDS	51.039	0.019	0.995	0.899	1.034	0.996
Fe_3_O_4_@CTAB	55.671	0.020	0.999	0.914	1.125	0.997
Fe_3_O_4_@SNC 16	52.362	0.021	0.999	0.909	1.122	0.997
Fe_3_O_4_@NPC 16	54.589	0.020	0.999	0.912	1.127	0.997

### 4.3. Regeneration experiments

**Figure 9.**
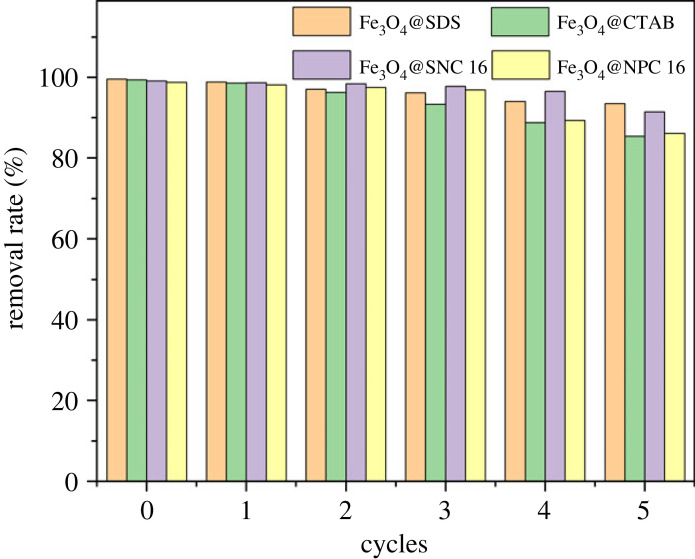
Recyling performance of the different Fe_3_O_4_@surfactants materials (pH 6, initial concentration 5 mg l^−1^, adsorbent dosage 1 mg ml^−1^, temperature 298 K, contact time 1 h).

According to Jin *et al*. [57], high pH drives down the adsorption of As(V) by Fe_3_O_4_@CTAB and alkali treatment is a feasible solution for desorption of As(V) from the adsorbent. The desorption rate remained constant when the NaOH concentration reached 1 mol l^−1^. Therefore, 1 mol l^−1^ NaOH was also used to desorb As(V) from the material in this experiment. After desorption, 0.01 mol l^−1^ HCl was used to wash the nanoparticles for 20 min and then washed with ultrapure water to neutral state. Figure 9 showed that approximately 85% removal capacity of As(V) was retained for the Fe_3_O_4_@surfactants even after five cycles, indicating excellent renewal potential of the adsorbent.

### 4.4. Post-cycle stability test

**Figure 10.**
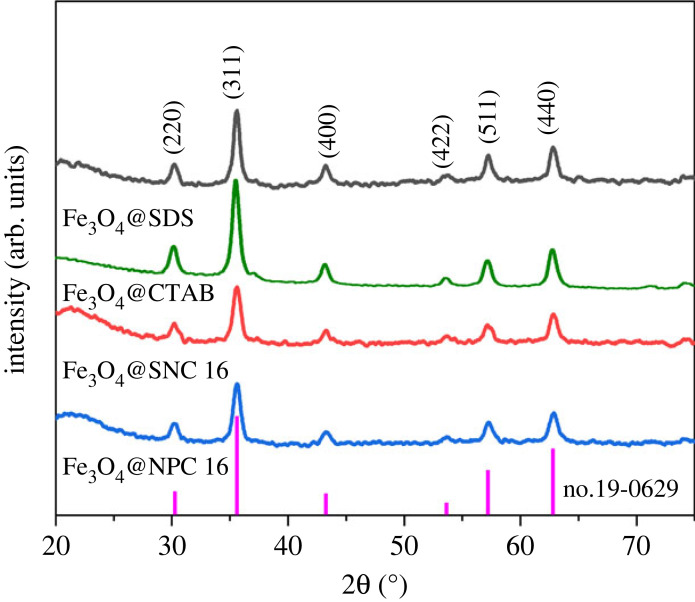
XRD plots of different Fe_3_O_4_@surfactants materials after five cycles (pH 6, initial concentration 5 mg l^−1^, adsorbent dosage 1 mg ml^−1^, temperature 298 K, contact time 1 h).

The XRD patterns of Fe_3_O_4_@surfactants after five cycles of regeneration are shown in figure 10. As shown in figure 10, the positions and relative intensities of the diffraction peaks as well as the crystal shapes of the Fe_3_O_4_@surfactants nanoparticles did not change after five cycles of adsorption–desorption, indicating that under the adsorption of As(V) and five cycles of regeneration, the Fe_3_O_4_@surfactants nanoparticles also have good stability. Overall, after the adsorption–desorption cycle, the Fe_3_O_4_@surfactants nanoparticles are stable and have good recycling performance in the removal of arsenic-containing wastewater.

## 5. Conclusion

The present study reported the synthesis of surfactant-modified Fe_3_O_4_ (Fe_3_O_4_@surfactants) for As(V) adsorption. A series of Fe_3_O_4_ morphologies are obtained due to the addition of surfactants. The average diameter of as-prepared Fe_3_O_4_@surfactants was about 10 nm with the high saturation magnetization values of 79.82 emu g^−1^. Batch equilibration studies showed stepwise adsorption of As(V) on these magnetic nanoparticles, which could be represented the best by the Langmuir equation. As estimated by the Langmuir model, the maximum As adsorption capacity of 55.671 mg g^−1^ can be obtained for Fe_3_O_4_@CTAB, being higher than those of the other type of surfactants. The adsorption kinetics followed the pseudo-second-order model and the experimentally obtained *E_a_* value indicates chemisorptions being probably the dominant factor in the adsorption process of As(V) onto surfactant-modified nanoparticles. From the comparative evaluation of different surfactant-modified magnetic nanoparticles, it was observed that the adsorption capacity of the adsorbents was closely related to the electrostatic interactions. The prepared Fe_3_O_4_-modified nanoparticles showed excellent arsenic removal effect from the water. The modified Fe_3_O_4_ nanoparticles have great potential in wastewater engineering for the removal of heavy metal ions.

## Data Availability

Data and relevant code for this research work are available from the Dryad Digital Repository: https://doi.org/10.5061/dryad.4f4qrfjg2 [59]. Data are provided in the electronic supplementary material [60].
